# Reversible Growth-Arrest of a Spontaneously-Derived Human MSC-Like Cell Line

**DOI:** 10.3390/ijms21134752

**Published:** 2020-07-03

**Authors:** Catharina Melzer, Roland Jacobs, Thomas Dittmar, Andreas Pich, Juliane von der Ohe, Yuanyuan Yang, Ralf Hass

**Affiliations:** 1 Biochemistry and Tumor Biology Lab, Department of Obstetrics and Gynecology, Hannover Medical School, 30625 Hannover, Germany; catharina.melzer@t-online.de (C.M.); Ohe.Juliane.von.der@mh-hannover.de (J.v.d.O.); kateyang-y@hotmail.de (Y.Y.); 2 Department of Rheumatology and Clinical Immunology, Hannover Medical School, 30625 Hannover, Germany; jacobs.roland@mh-hannover.de; 3 Institute of Immunology, Center for Biomedical Education and Research (ZBAF), Witten/Herdecke University, 58453 Witten, Germany; thomas.dittmar@uni-wh.de; 4 Department of Toxicology, Hannover Medical School, 30625 Hannover, Germany; pich.andreas@mh-hannover.de

**Keywords:** mesenchymal stroma/stem cells, MSC marker, MSC cell line, reversible growth arrest, tissue regeneration

## Abstract

Life cycle limitation hampers the production of high amounts of primary human mesenchymal stroma-/stem-like cells (MSC) and limits cell source reproducibility for clinical applications. The characterization of permanently growing MSC544 revealed some differentiation capacity and the simultaneous presence of known MSC markers CD73, CD90, and CD105 even after continuous long-term culture for more than one year and 32 passages. The expression of CD13, CD29, CD44, and CD166 were identified as further surface proteins, all of which were also simultaneously detectable in various other types of primary MSC populations derived from the umbilical cord, bone marrow, and placenta suggesting MSC-like properties in the cell line. Proliferating steady state MSC544 exhibited immune-modulatory activity similar to a subpopulation of long-term growth-inhibited MSC544 after 189d of continuous culture in confluency. This confluent connective cell layer with fibroblast-like morphology can spontaneously contract and the generated space is subsequently occupied by new cells with regained proliferative capacity. Accordingly, the confluent and senescence-associated beta-galactosidase-positive MSC544 culture with about 95% G0/G1 growth-arrest resumed re-entry into the proliferative cell cycle within 3d after sub-confluent culture. The MSC544 cells remained viable during confluency and throughout this transition which was accompanied by marked changes in the release of proteins. Thus, expression of proliferation-associated genes was down-modulated in confluent MSC544 and re-expressed following sub-confluent conditions whilst telomerase (hTERT) transcripts remained detectable at similar levels in both, confluent growth-arrested and proliferating MSC544. Together with the capability of connective cell layer formation for potential therapeutic approaches, MSC544 provide a long term reproducible human cell source with constant properties.

## 1. Introduction

Tissue-derived primary mesenchymal stroma-/stem-like cells (MSC) represent a heterogeneous cell mixture comprised of multiple types of distinct stroma and stem-like cells rather than a uniform population. Accordingly, MSC exhibit therapeutic potential associated with multiple and controversially discussed functions. These include early developmental tasks, and later on in adult human tissues, distinct repair and regenerative activities, involvement in neovascularization, immune-modulatory capabilities, paracrine effects, antimicrobial functions, tumor-inhibitory and tumor-promoting properties, and cell fusion, among others. Thus, MSC are attributed with different nomenclatures like mesenchymal stromal or mesenchymal stem(-like) or medicinal signaling cells [1,2]. These often-not-precisely but interchangeably used terms underscore the still limited understanding about characteristics and the biological role of MSC [3]. The characteristics of MSC can vary in a changing environment by diverse stimuli and culture conditions in vitro or according to the different originating tissues in vivo. Thus, human MSC can be derived from neonatal birth-associated tissues such as the placenta (PL-MSC), umbilical cord (UC-MSC), and amniotic membrane (AM-MSC), and may exhibit superior in vitro growth potential and regenerative capacity as compared to adult tissue-derived MSC including peripheral blood (PB-MSC), bone marrow (BM-MSC), or adipose tissue (AD-MSC (also termed AT-MSC or ASC)) [3,4].

Considering their heterogeneity, MSC are characterized by minimal criteria, like in vitro plastic adherence, migratory activity [5], differentiation along mesenchymal phenotypes, distinct surface marker expression [6,7] and specific stem cell features such as self-renewal capacity. However, these characteristics may not equally apply to all MSC within a population which enables discrimination of subpopulations. Thus, a small subpopulation of stem(-like) cells with certain differentiation capacity and proliferative self-renewal potential can be part of a panel of other stromal subpopulations lacking these features but displaying the remaining MSC characteristics. Consequently, we used the term mesenchymal stroma-/stem-like cells to acknowledge and distinguish these subpopulations within a heterogeneous MSC entity.

The variety of MSC subpopulations with different functionalities is further complexed by the problem of discriminating these cells from closely related cell types, including fibroblasts and pericytes. While pericytes display multi-lineage potential in vitro, these cells were also discussed to contribute to tissue repair and regeneration by demonstrating similar properties like MSC [8,9,10]. However, previous studies suspected potential cell culture-mediated alterations responsible for such in vitro plasticity since pericytes from several adult tissues such as skeletal muscle, adipose tissue, brain, and heart, may not represent multipotent tissue-resident progenitors suggesting pericytes a more differentiated population. As a further discrimination factor, these pericytes, besides vascular smooth muscle cells, are reported for their selective expression of the transcriptional repressor Tbx18 (T-box transcription factor) involved in the regulation of developmental processes [11].

Expression of telomerase may represent an additional feature for the identification of a stem-like subtype within a stromal population. Active human telomerase reverse transcriptase (hTERT) for the protection of genomic DNA by safeguarding chromosomal end replication with telomeric repeats is primarily found in stem cells and immortal neoplastic cells to ensure continued cell cycle progression. In contrast, progressive telomeric erosion by hTERT inhibition/down-modulation is observed in growth-arrested somatic cells and during either stress-induced or replicative senescence [12]. In primary human MSC, the expression of telomerase persists during prolonged culture until these cells progressively enter senescence (e.g., beyond P7 to P10 in UC-MSC cultures) suggesting a limited maintenance of a stem-like subpopulation within the MSC culture [13].

To date, studies with human MSC are performed in individual primary cultures with an overall limited availability, reproducibility, and life span. Consequently, a human cell line with appropriate MSC-like characteristics providing unlimited growth potential and reproducible characteristics could serve as a valuable uniform model, e.g., for a constant cell source in regenerative medicine or the identification of certain MSC subtypes developing during changed environmental conditions.

The present work characterizes permanently growing human MSC544 with a constitutive expression of typical MSC-like markers including immune-modulatory functions. Moreover, MSC544 can display an alternating reversible transition between a growth-arrest and a proliferative state providing a representative model for studying subpopulation development and reproducible regenerative potential. Thereby, this study also extends the panel of further markers for the characterization of different MSC populations. 

## 2. Results 

**Figure 1:**
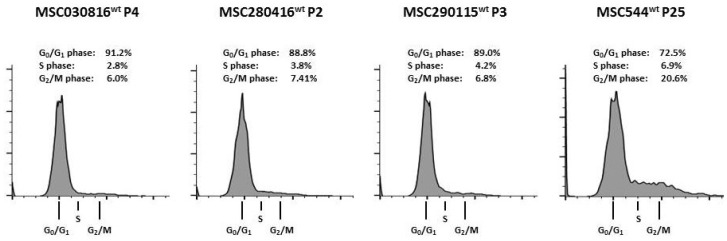
Cell cycle analysis of three different primary human umbilical cord-derived (UC)- mesenchymal stroma-/stem-like cell (MSC) populations in steady state at low passages were compared to the cell cycle progression of MSC544 in steady state at passage 25.

Primary human MSC populations derived from several explant cultures of three different umbilical cord tissues in passage (P)2 to P4 demonstrated a consistent steady state cell cycle progression, displaying about 89% to 91% of cells in G0/G1 phase, about 3% to 4% in S phase, and about 6% to 7% in G2/M phase. In contrast, the neoplastic tissue-derived MSC544 in P25 at similar culture conditions with a constitutive doubling time of about 30h demonstrated a markedly increased presence of cells in the proliferative cell cycle phases including about 7% in S phase and about 21% in G2/M phase while about 73% were detectable in G0/G1 phase (Figure 1).

The characterization of MSC544 for distinct protein markers by flow cytometry and comparison to 12 different MSC populations in vitro derived from six umbilical cords, three placentae, and three bone marrows revealed distinguishable subpopulations in the FSC/SSC analysis of MSC544 but all with similar marker expression levels (Table 1). Beyond the minimal MSC markers CD73, CD90, and CD105, we additionally found the presence of the membrane-associated alanyl aminopeptidase (EC 3.4.11.2) CD13, the fibronectin receptor β-subunit β1 integrin CD29, the hyaluronan receptor CD44, and the activated leukocyte cell adhesion molecule (ALCAM) transmembrane glycoprotein CD166 in nearly all MSC tested (Table 1). These findings add further common functionalities to the autonomously proliferating MSC544 as compared to MSC populations derived from the different tissues by including regulatory enzyme activity for peptide metabolism (CD13) and distinct cell communication and matrix receptor proteins (CD44, CD166, CD29). 

Whereas further common proteins involved in cellular communication were detectable in the different MSC cultures investigated, like the transmembrane cancer-antigen 125 (=mucin 16) glycoprotein CA125, the melanoma cell adhesion molecule (=mucin 18) CD146, the intercellular adhesion molecule-1 CD54, the vascular cell adhesion molecule-1 CD106, and the glycophosphatidylinositol-anchored cell membrane protein mesothelin, these markers were only partially expressed in various cell types and may therefore represent MSC subpopulations within the whole cultures (Table 1). Indeed, previous work suggested that, e.g., CD146-positive MSC from different tissues display altered transcriptional profiles and differentiation capacities [14].

Only little if any expression was detectable for other surface marker proteins, some of which serve as simultaneous exclusion criteria for MSC including the endothelial marker CD31, the monocyte marker CD14, or the lymphocyte marker CD45 among others (Table 1, markers with khaki background). Together, these data demonstrated typical mesenchymal stroma-/stem cell-like characteristics of MSC544 by extending the in vitro characterization of a MSC phenotype which could contribute to further discriminate potential subpopulations.

Functional characteristics of MSC include differentiation capacity and immune-modulatory capabilities which were tested in MSC544. Upon culture in appropriate differentiation media, MSC544 demonstrated some maturation along the osteogenic lineage by enhanced Alizarin red staining and chondrogenic features by elevated Alcian Blue staining. The stimulation of adipogenic differentiation in MSC544 revealed a slight induction of the fatty acid-binding protein 4 (FABP4) and the transcription factor CCAAT/enhancer-binding protein alpha (CEBPα) after normalization to β-actin (Supplementary Figure S1).

**Figure 2:**
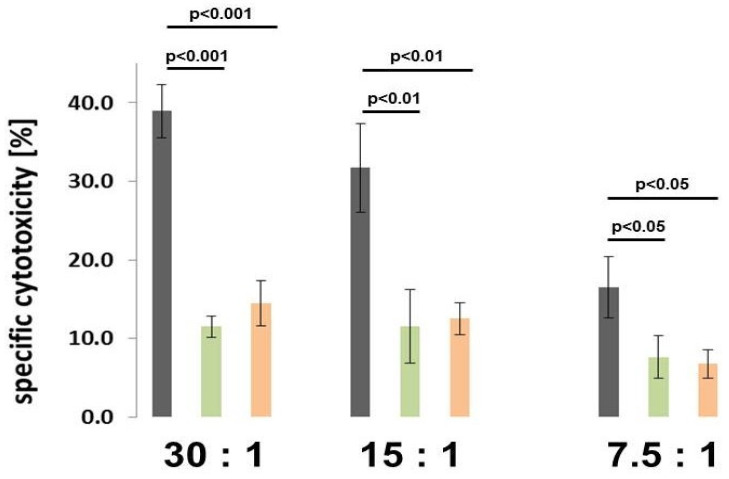
Immune-modulatory activity was tested with peripheral blood mononuclear cells (PBMCs) following stimulation by different MSC544 subpopulations. PBMCs as effector cells alone (gray columns) and after co-culture together with proliferating MSC544 P27 in steady state (green columns) or with growth-arrested confluent MSC544 after permanent culture for 189d (orange columns) were incubated with ^51^Cr–labeled K652 target cells at three different effector to target (E:T) ratios and released radioactivity was determined from cell-free supernatants. Minimal (supernatant from samples without effector cells) and maximal radioactivity values (supernatant from detergent-lysed target cells) were used for calculating the percentage of specific cytotoxicity of the effector cells. Data represent the mean ± s.d. (*n* = 3) and significance (*p*) was evaluated by paired Student’s-*t*-test.

MSC display regulatory effects on the innate and adaptive immune system although precise functions of this cellular crosstalk still remain a matter of debate [15]. Accordingly, immune-modulatory capabilities of MSC544 were tested in vitro with peripheral blood mononuclear cells (PBMCs). Due to the autonomous growth properties of MSC544, we were also interested as to whether growth-arrested subpopulations of MSC544 could exhibit potential immune-modulation. Therefore, MSC544 were cultured to confluency and maintained at this growth-arrested state for further 189d by only replacing the culture medium once a week. After the stimulation of freshly-isolated PBMCs by either proliferating MSC544 in P27 or by growth-arrested confluent MSC544 in P22 after continuous culture for further 189d, the PBMCs as effector cells were co-cultured with ^51^Cr–labeled K652 target cells to monitor cytotoxicity of target cell killing activity. The percentage of specific cytotoxicity was calculated via detection of cell-free ^51^Cr radioactivity in the supernatant and revealed a marked down-modulation of PBMC killing activity under the influence of both, proliferating and growth-arrested confluent MSC544. Compared to non-stimulated PBMCs as a control (Figure 2, gray bars) with specific cytotoxicity of 38.9% + 3.4% using an effector to target cell ratio of 30:1, the specific cytotoxicity significantly declined by 3.4-fold to 11.5% + 1.3% following stimulation with proliferating MSC544 P27 (Figure 2, green bars). Likewise, specific cytotoxicity was significantly inhibited by 2.7-fold down to 14.5% + 2.9% after incubation of PBMC with growth-arrested confluent MSC544 P22 (Figure 2, red bars). Similar effects were obtained after using an effector to target cell ratio of 15:1 and 7.5:1, respectively. Moreover, the data demonstrated no significant differences between the immune-modulatory capacities of proliferating MSC544 P27 versus growth-arrested confluent MSC544 P22. 

**Figure 3:**
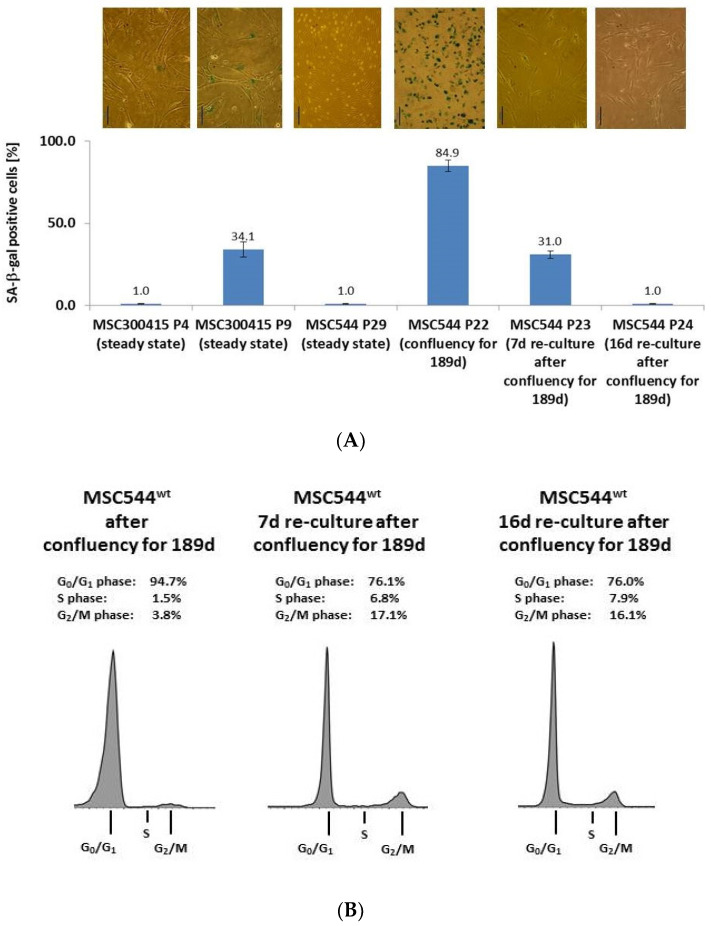
(**A**) Expression of senescence-associated beta galactosidase (SA-β-gal) was monitored for enzymatic activity after processing the substrate 5-bromo-4-chloro-3-indolyl-βD-galactopyranoside to obtain blue colored cells via light microscopy (upper panel, bars represent 100µm). These were quantified by the percentage of SA-β-gal-positive cells in UC-MSC300415 in a low (P4) and high (P9) passage compared to MSC544 in P29. Moreover, SA-β-gal expression was evaluated in MSC544 P22 grown in confluency for 189d without subculturing and compared to MSC544 grown in confluency for 189d and subcultured for additional 7d in P23 and 16d in P24, respectively. Data represent the mean + s.d. (*n* = 3). (**B**) Cell cycle analysis was performed in MSC544 P22 grown in confluency for 189d without subculture and compared to MSC544 grown in confluency for 189d and subcultured for additional 7d in P23 and 16d in P24, respectively.

Whereas MSC544 display stroma-/stem cell-like characteristics with permanent proliferative capacity, we further addressed the question what happens to MSC544 in a confluent environment and then after passage and re-culture at normal subconfluent conditions. Previous work has documented that normal primary human UC-MSC maintain proliferative capacity only for up to about 10 cell passages during in vitro culture until senescence [13,16,17]. This was substantiated by quantification of senescence-associated β-galactosidase (SA-β-gal) activity in proliferating primary UC-MSC cultures with about 1% of SA-β-gal activity in P5 as compared to about 34% SA-β-gal in cells of P9 (Figure 3A). Permanently growing MSC544 in steady state culture displayed about 1% of SA-β-gal activity in P29. In contrast, MSC544 P22 after 189d of permanent culture in confluency displayed about 85% of SA-β-gal positive cells. Following subculture of this growth-arrested population in a normal subconfluent environment, however, the amount of SA-β-gal positive cells significantly declined to about 31% after 7d and further dropped to about 1% after 16d of re-culture (Figure 3A).

The SA-*β*-gal expression levels in the different MSC544 populations were paralleled by corresponding cell cycle data. The 189d confluent MSC544 population demonstrated about 95% cell cycle arrest in G0/G1 phase. However, a 7d reculture of the whole confluent culture at subconfluent conditions revealed reentry into the cell cycle by a decrease of G0/G1 phase cells down to about 76% and a corresponding increase of S phase and G2/M phase cells to about 7% and 17%, respectively, which was similarly observed after 16d of re-culture (Figure 3B). Of interest, little—if any—significant appearance of an apoptotic/necroptotic subG1 population was detectable after resumed proliferation from the growth-arrested state.

The maintenance of differently-shaped green fluorescent protein (GFP)-labeled MSC544 in steady state (Supplementary Figure S2A) during long-term culture in a confluent state was associated with a progressive change in morphology by developing a spindle fibroblast-like phenotype and expression of a stable extracellular matrix to connect the confluent cells in a common tissue-like layer (Supplementary Figure S2B). This dense layer of connected cells then spontaneously started circular detachment from the culture dish at some places by simultaneous contraction and subsequent disruption of the cell layer (Supplementary Figure S2C,D) leaving some disrupted bodies of cell fragments (Supplementary Figure S2E) and building a dense structure of stroma-like tissue (Supplementary Figure S2F). After disruption and contraction of the tissue-like layer, small heterogeneously-shaped MSC544 started to proliferate again in the regained cell-free areas (Supplementary Figure S2G). 

**Figure 4:**
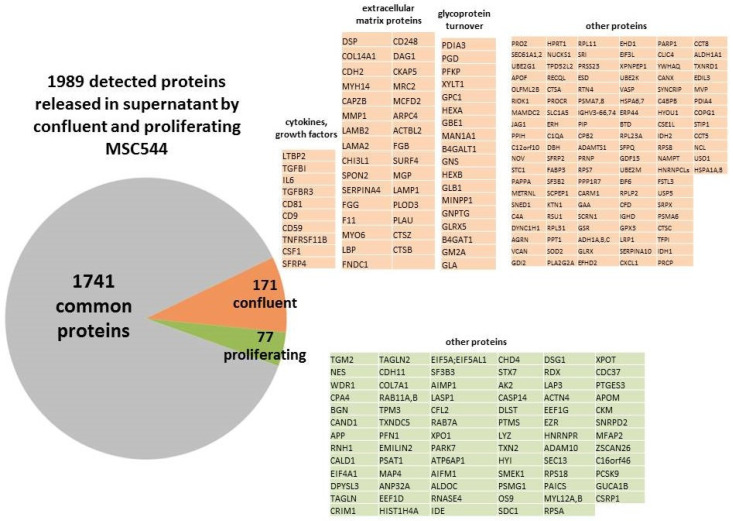
Proteomics analysis was performed by LC-MS of 36h cell culture supernatant following secretion of proteins from steady-state proliferating MSC544 in comparison to a corresponding 36h release of proteins from confluent MSC544 after permanent culture for 189d. Gene names of the proteins are presented in the tables whereby 171 secreted proteins identified from the confluent MSC544 cell culture (orange tables) were further distinguished by functional groups with little if any corresponding functionalities in the released proteins from proliferating MSC544 (green table).

These morphological changes during long-term culture and the transition from a proliferative steady state culture to a growth-arrested confluent phenotype is determined by an altered environment and requires functional changes in gene and protein expression and release. Consequently, we performed proteome analysis of factors released into the medium by an equal cell number after 36h (Figure 4). This conditioned medium from proliferating and confluent MSC544 revealed 1989 proteins detectable by LC/MS analysis from which 248 were differentially expressed. The majority of 171 proteins was released by 189d confluent MSC544 but undetectable in the supernatant of proliferating MSC544. Vice versa, only 77 proteins released by proliferating MSC544 remained below detection limit in the 189d confluent MSC544 conditioned medium. In contrast to proliferating MSC544 different cytokines and growth factors including tumor necrosis factor-associated proteins, interleukin-6, transforming growth factor-beta and macrophage colony-stimulating factor-1 were released by confluent MSC544 as well as tetraspanins (CD9, CD81) which are associated with extracellular vesicles such as exosomes (Figure 4).

In addition, a variety of proteins released by confluent MSC544 were identified as structural extracellular matrix proteins (e.g., collagens, cadherins, laminins) and enzymes which reorganize the extracellular matrix (e.g., cathepsins, plasminogen activators) and the glycosylation pattern suggesting enhanced matrix structures and increased turnover of glycoproteins in the extracellular space of confluent MSC (Figure 4).

**Figure 5:**
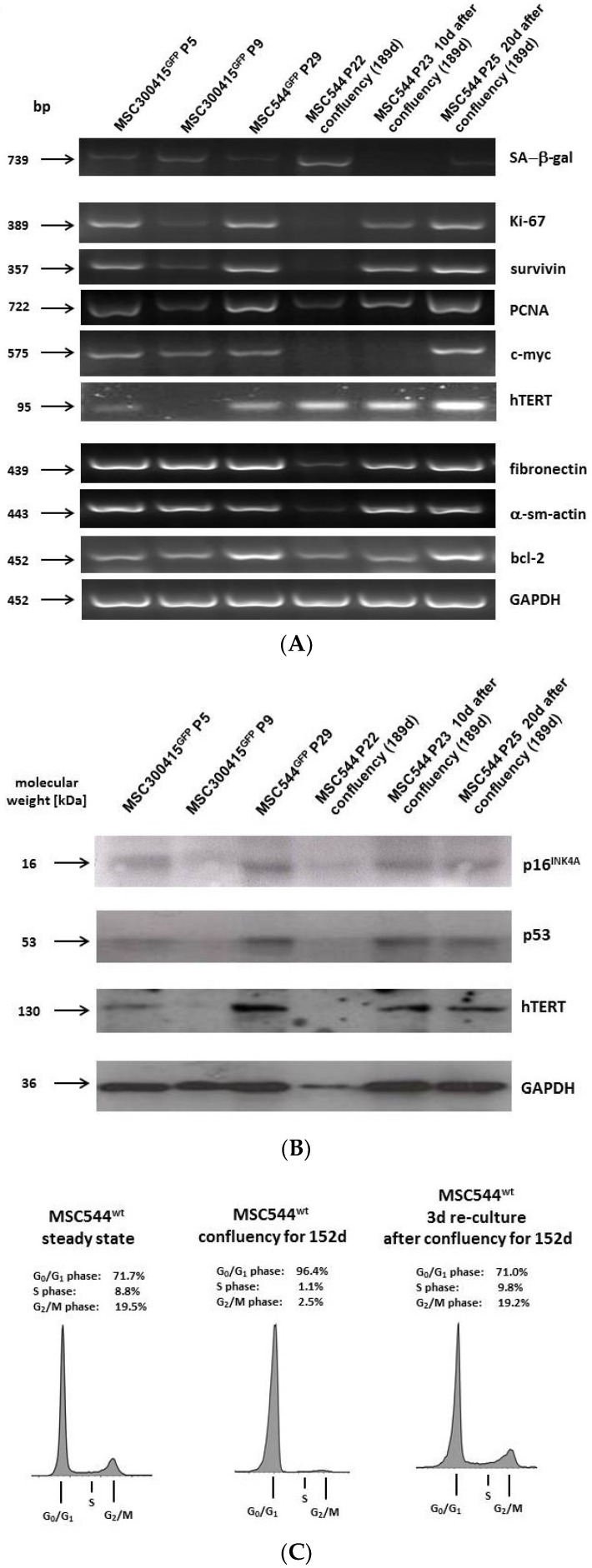

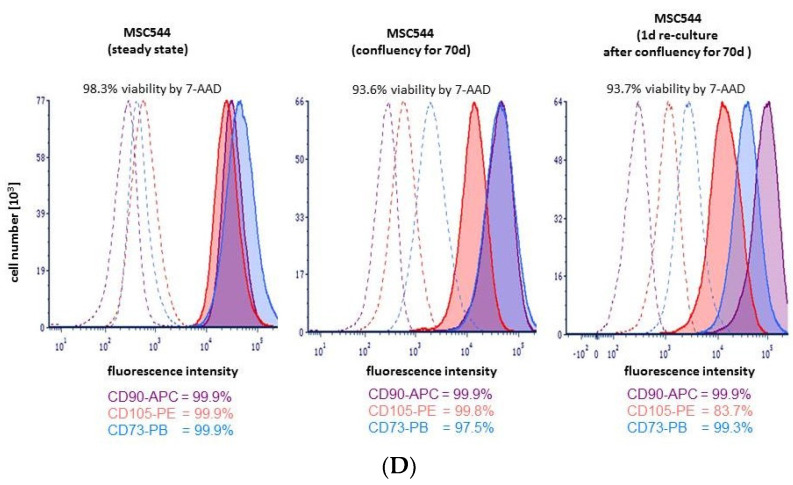
(**A**) Expression pattern of distinct genes was determined by RT-PCR in UC-MSC300415 in a low (P5) and high (P9) passage. Moreover, mRNA transcripts were evaluated in MSC544 P22 grown in confluency for 189d without subculture and compared to MSC544 grown in confluency for 189d and subcultured for additional 10d in P23 and 20d in P25, respectively. (**B**) Protein expression of p16^INK4A^, p53, and human telomerase reverse transcriptase (hTERT) in UC-MSC300415 in a low (P5) and high (P9) passage was determined by Western blot analysis. Moreover, protein levels were evaluated in MSC544 P22 grown in confluency for 189d without subculture and compared to MSC544 grown in confluency for 189d and subcultured for additional 10d in P23 and 20d in P25, respectively. Expression of GAPDH was used as a control. (**C**) Cell cycle analysis of steady state MSC544 was compared to 152d continuously confluent MSC544 and after subconfluent re-culture of 152d growth-arrest MSC544 for 3d. (**D**) Simultaneous measurement of CD90-APC, CD105-PE, and CD73-PB by flow cytometry in steady state MSC544 as compared to MSC544 cultured for 70d in confluency and 70d confluent MSC544 after 1d re-culture in a sub-confluent environment. Viability of the cultures was assessed by 7-AAD staining.

These differences in the functionality of proliferating and growth-arrested confluent MSC544 was also reflected by a selected transcript pattern (Figure 5A). Enhanced expression of SA-β-gal was detectable in primary UC-MSC300415 P9 and particularly in 189d confluent MSC544. Vice versa, little if any transcripts of proliferation-associated genes including Ki67, survivin, proliferating-cell-nuclear-antigen (PCNA), and c-myc were detectable in the 189d growth-arrested MSC544 in contrast to their proliferating counterparts. Of interest, human telomerase reverse transcriptase (hTERT) mRNA levels were only down-modulated in primary UC-MSC300415 P9 but remained unchanged in both, proliferating MSC544 and in the 189d senescent MSC544 (Figure 5A). While the matrix transcripts fibronectin and α-smooth muscle actin (α-sm-actin) demonstrated lower expression levels in the 189d confluent MSC544 the anti-apoptotic bcl-2 was pronounced expressed and GAPDH transcripts served as loading control (Figure 5A).

A more detailed analysis of hTERT and of the DNA stability-sensing and senescence-related proteins p16^INK4A^ and p53 was performed at the protein level by Western blot analysis and revealed little if any protein expression in primary UC-MSC300415 P9 and the 189d SA-β-gal-positive MSC544 although GAPDH expression was lower in the confluent MSC544 cultures. In contrast, a prominent p16^INK4A^, p53, and hTERT protein expression was detectable in all other proliferating MSC cultures (Figure 5B). Whereas SA-β-gal expression is not limited to senescence but also observed in confluency and oxidatively-stressed cells [18] a paralleled down-modulation of p16^INK4A^ and p53 in the growth-arrested MSC544 population may further contribute to a phenotype of replicative senescence [19]. p16^INK4A^ affects activation of the retinoblastoma protein (pRB), specifically by inhibiting cyclin-dependent kinases among further pathways. However, chromatin restructure with cell cycle re-entry and resumed proliferation can be regulated by completely different expression levels of p16^INK4A^ as demonstrated, e.g., in human BJ foreskin fibroblasts versus human WI-38 lung fibroblasts. In parallel, alterations in p53 expression and associated signaling cascades can trigger reversal of growth arrest and cellular senescence in human cells [20].

The velocity of MSC544 transition from a growth-arrested confluent state back to a proliferative active population was determined in additional long-term culture experiments and occurred rapidly since the 152d confluent MSC544 population with 96% G0/G1 growth arrest returned to a normal cell cycle progression of about 71% G0/G1, 10% S phase, and 19% G2/M phase within 3d after transfer to subconfluent conditions which was similar to the cell cycle distribution observed in steady state MSC544 (Figure 5C).

Moreover, steady state MSC544 after transition to a growth-arrested confluent cell layer for 70d and back to subconfluent proliferating conditions exhibited persisting MSC properties by prominent simultaneous expression of the three MSC markers CD73, CD90, and CD105 (Figure 5D). Analysis by 7-AAD staining revealed more than 90% of viable cells in all three MSC544 populations (Figure 5D) which is in agreement with Figure 3B demonstrating no detectable appearance of apoptotic/necroptotic subG1 populations. 

These findings suggested little if any dying cells in a senescent state of MSC544. In contrast, the MSC544 maintained viability during transition to a growth-arrested state and vice versa back to proliferative conditions.

Analysis of additional surface markers likewise revealed no significant differences in the protein expression levels of the three MSC544 populations except for CD54 (Table 2). This intercellular adhesion molecule-1 was expressed by about 25% in steady state MSC544, increased to about 91% in the dense population of confluency and thereafter, dropped to about 77% after 1d re-culture in a subconfluent environment (Table 2). These findings suggested that CD54 expression may be associated at least in part with the density of the MSC544 population and a corresponding amount of cell-cell interactions. Indeed, a 3d re-culture at subconfluent conditions with a switch to fewer cell-cell interactions and more extracellular matrix interactions was associated with a further decline in CD54 expression to about 56%.

Further MSC544 characterization was performed for a cell line authentication pattern by short tandem repeat (STR) fragment analysis. Comparison of two morphological and functional subpopulations including proliferating MSC544 P16 and growth-arrested MSC544 P25 after 152d of continuous confluency revealed identical STR patterns (Supplementary Figure S3).

Although this MSC544 cell line with the potential of reversible switch between growth-arrested and proliferative state represents an excellent in vitro model also for studying tissue replacement and regenerative potential in a translational view, consideration should be given to the fact that MSC544 were originally derived from a patient with phalloid tumor. However, in vivo studies (n = 5) were scored for different parameters and revealed no detrimental or unhealthy development/animal behavior:


  burden:						none (5/5)
  body condition:				very good (5/5)
  behavior:						very active (5/5)
  subcutaneous injection site:		no abnormalities (5/5)
  spleen and liver:				no abnormalities (5/5)
	  

In sum, there was no detectable malignant development or tumor growth in any of the five immune-deficient mice after subcutaneous injection of 10^6^ MSC544 and observation for the following 98 days.

## 3. Discussion

Normal primary human MSC maintain proliferative capacity, cell fate, and marker expression for a limited number of cell passages during in vitro culture [13,16,17]. In contrast, the neoplastic tissue-derived MSC544 continued to grow and maintained expansion potential and the expression of MSC markers beyond this limitation.

The typical cell surface molecules CD73, CD90, and CD105 as minimal characteristics for the identification of MSC were detectable in primary MSC from the umbilical cord, bone marrow, and placenta at low passages (P0 to P4), whereby similar expression levels in MSC544 also confirmed MSC-like properties in a high passage of P22 [21]. This substantiated a long-term presence of typical MSC markers in continuously growing MSC544 since previous in vitro lineage tracing work demonstrated that primary MSC beyond passage 9 to 10 progressively lose these markers [13,16]. The panel of surface molecules was enlarged by CD13, CD29, CD44 and CD166 which is in line with previous characterizations of BM-MSC and AD-MSC, except for Stro-1 [22]. More partially expressed proteins including CD54 and CD146 in the tested MSC cultures suggest certain subpopulations within the MSC entity according to the corresponding culture conditions applied. The appropriate plasticity of marker protein expression by potential mixtures of distinct subpopulations displaying mutually dependent and adaptive expansion properties has also been suggested for the development of MSC/cancer hybrid cells after fusion and putatively arising stem cells [23]. Thus, a combination of partially expressed markers together with other generic MSC markers could provide discrimination criteria of stroma-/stem cell-like subpopulations. For example, CD146-positive cells with MSC-like features in the bone marrow were characterized as hematopoiesis-supporting Angiopoietin-1-expressing osteoprogenitors displaying in vivo self-renewal capacity consistent with stem cell-like properties [24]. 

Typical morphology, surface marker expression, differentiation properties, and persistent telomerase transcripts supported mesenchymal stroma-/stem-like properties of MSC544. With respect to the acquisition of some differentiation properties MSC544 may also exhibit alternative maturation pathways, e.g., fibroblast-like differentiation after confluency. The observed differentiation parameters can be limited by the in vitro environment since, e.g., confluency-promoted mechanical stress or the rigid plastic substrate favorises osteogenic maturation in contrast to soft gels which facilitate MSC differentiation along the adipogenic lineage [25,26]. However, in vitro differentiation may include artificial parameters and significantly differs from MSC differentiation in vivo. Thus, in contrast to in vitro studies, previous studies have demonstrated in vivo that platelet-derived growth factor receptor alpha-expressing adventitial or capsular fibroblasts rather than MSC represent the predominant adipocyte precursor population which contributes to brown, beige, and white adipocyte differentiation [27]. 

Immune-modulatory capacity substantiated a mesenchymal stroma-/stem cell-like phenotype of MSC544 by significantly reducing the cytotoxic capacity of PBMCs. This effect is contributed by the intracellular heme-containing enzyme indoleamine-2,3-dioxygenase converting tryptophan to N-formylkynurenine [28]. In addition, arachidonic acid metabolism paralleled by appropriate cyclooxygenase activity and subsequent prostaglandin E2 production further supports MSC immune-suppressive properties.

The reversible transition between proliferating and growth-arrested MSC544 populations provides an interesting model for testing different stimuli and culture conditions with respect to selection and identification of subsequent subpopulations and a reversible subpopulation switch. Longer-term culture of MSC on distinct soft or stiff substrates provides population stringency with narrowing lineage convergence [29,30]. A uni-directional subpopulation switch was also observed in normal UC-MSC cultures since our previous work using bar code vector-labeling demonstrated a significant reduction in diversity and simultaneous clonal convergence of UC-MSC during subsequent passages and reduced proliferative capacity [31]. Similar effects may apply to MSC544 during cell cycle arrest in a confluent environment. Conversely, transition from confluency-mediated growth arrest back to cell cycle reentry could progressively increase heterogeneity of distinct subpopulations with interdependent growth and survival properties as suggested for post-fusion selection of MSC/cancer hybrid cells [23]. However, a possible clonal convergence during G0/G1 cell cycle arrest of confluent and growth-arrested MSC544 followed by transition back to a proliferative state in a sub-confluent environment with mutually dependent and adaptive expansion properties remains to be elucidated. Whereas reversible growth arrest of MSC544 is observed without detectable cell death, apoptotic/necroptotic cells may disappear from the cell layer by rapid disintegration of cellular parts into the supernatant. Alternatively, apoptotic cells could be eliminated from the culture by engulfment since previous work has documented the capability of MSC for clearance of apoptotic cells [32]. At the very least, potential engulfment or entosis demonstrated no detectable differences in the DNA pattern of the STR fragment analysis by comparing proliferating and growth-arrested MSC544.

Progressively closer interactions of MSC544 with the matrix by the various adhesion molecules built a connected cell layer of a confluent growth-arrested MSC544 culture. Previous work suggested that prolonged MSC culture on stiff surfaces like the negatively-charged polystyrene dishes is accompanied by loss of MSC markers, osteogenic maturation and replicative senescence which can be reverted by culture on soft hydrogel matrices [33]. Although matrix factors in MSC544 such as fibronectin and stress fiber-associated α-sm-actin demonstrated low mRNA transcripts, proteins of other extracellular matrix components including collagens, N-cadherins, and laminins were markedly detectable in confluent MSC544 cultures in contrast to their proliferating counterparts. Cytoskeletal movement forces supported by acto-myosin motility can trigger differentiation processes [29] whereby MSC544 displayed the formation of a fibroblast-like phenotype together with a subsequent matrix-connected cell layer consisting of tightly attached spindle-like cells. In parallel to the matrix generation and alterations in the glycosylation patterns, certain matrix-digestive enzymes spontaneously lead to a detachment with subsequent progressive contraction of the whole connected MSC544 layer. Indeed, previous data substantiated that different MSC populations can release extracellular matrix-restructuring enzymes such as various matrix metalloproteinases and urokinase-type plasminogen activator [21]. The spontaneous contraction of matrix with MSC544 cell layers, however, was paralleled by local restoration of proliferative potential whereby small heterogeneously-shaped MSC544 refilled the available space in the culture dish up to contact inhibition by another confluent cell layer. These findings indicated reversible proliferative capacity paralleled by certain regenerative potential.

Moreover, in vivo data substantiated no neoplastic or cancerous development as evaluated by healthy animal behavior for more than three months after transplantation of proliferating MSC544 suggesting no detectable tumorigenicity. These findings may eventually provide MSC544 as a vehicle with off-the-shelf therapeutic potential.

Together, the introduction of new populations such as the permanently growing human MSC544 displaying mesenchymal stroma-/stem cell-like properties provides a basis for further characteristic insights to further the limited knowledge about different tissue-derived and heterogeneous MSC variants. Consequently, the cell surface markers CD13, CD29, CD44, CD166, partially expressed proteins such as CD54 and CD146, and functional potency assays paralleled by distinct molecular patterns may serve as additional MSC markers contributing to a better identification of MSC heterogeneity and subpopulations in vitro. Moreover, high CD54 expression levels like in confluent MSC544 which can facilitate engraftment and homing to injured or damaged tissues may provide a preferred vehicle for transplantation in cell-based therapies [34].

## 4. Materials and Methods 

### 4.1. Cell Culture

Explant cultures for enrichment of primary human mesenchymal stroma/stem-like cells (MSC) was performed as described for umbilical cord tissue (UC-MSC) [35] and for placenta (PL-MSC) [4]. Moreover, primary human MSC were isolated from bone marrow (BM-MSC) according to a previous protocol [36]. In addition, primary MSC544 were derived from mammary tissue explants of a patient with a benign phyllodes tumor and isolation of cells from neoplastic breast tissue was described elsewhere [37]. Briefly, collected tissue was cut into small pieces of approximately 1 mm^3^ and extensively washed with PBS to remove blood cells and cell debris. The tissue pieces were incubated for explant culture and outgrowing cells revealed a predominant MSC-like morphology which were cultivated further at MSC-like growth conditions. The different MSC populations were cultured in MSC growth medium (αMEM (Sigma Chemie GmbH, Taufkirchen, Germany) supplemented with 10% allogeneic human AB-serum (blood from 31 male AB donors was commercially obtained from blood bank, Hannover Medical School, Germany, and processed to serum), 100 U/mL penicillin, 100 µg/mL streptomycin and 2 mM L-glutamine (Sigma Chemie GmbH) at 37 °C with 5 % CO_2_ in a humidified atmosphere. Subculture in passages (P) was performed following treatment of the different MSC cultures with accutase (Capricorn Scientific GmbH, Ebsdorfergrund, Germany) and treatment of MSC544 using TrypLE (Life Technologies GmbH, Darmstadt, Germany) at 37°C for 3 min. The study was conducted in accordance with the Declaration of Helsinki, and the use of human primary cells was approved by the Ethics Committee of Hannover Medical School, project #3916 on June 15th, 2005 and project #443 on February 26th, 2009 whereby informed written consent was obtained from each patient. For the experiments, MSC cultures were used from thirteen different donors (6x UC, 3x PL, 3x BM, and MSC544) in different passages: 

UC-MSC180816 P0, UC-MSC270815 P0, UC-MSC030816 P1, UC-MSC151116 P1, UC-MSC210611 P2, UC-MSC040211 P3,

BM-MSC84 P2, BM-MSC161115-I P3, BM-MSC161115-2 P4,

PL-MSC250116 P1, PL-MSC270116 P1, PL-MSC210116 P2,

and MSC544 P22 to P29

The different populations were tested for mycoplasma by the luminometric MycoAlert Plus mycoplasma detection kit (Lonza Inc., Rockland, ME, USA) according to the manufacturer’s recommendations.

Stable transduction of MSC544 cells for morphological discrimination in culture was performed with a 3rd generation lentiviral SIN vector carrying the enhanced green fluorescent protein (eGFP) gene [38]. 

### 4.2. Flow Cytometry Analysis

Analysis of the different MSC samples by flow cytometry was performed as described [39]. After blocking with 2 % Fetal calf serum (FCS) for 15 min at room temperature and subsequent washing in PBS the cells were stained with 7-AAD (7-aminoactinomycin D) viability staining solution (BioLegend via Biozol GmbH, Eching, Germany) and the following monoclonal anti-human APC- (allophycocyanin), PB- (pacific blue), PE- (phycoerythrin) or FITC- (fluorescein isothiocyanate) labeled antibodies: CD73-PE and CD73-PB (clone AD2, BD Bioscience GmbH, Heidelberg, Germany); CD90-PE and CD90-APC (clone 5E10, BioLegend via Biozol GmbH); CD105-PE (clone 43A3, BioLegend via Biozol GmbH); rat CD146-PE (clone ME-09F1, Miltenyi Biotech GmbH, Bergisch Gladbach, Germany); CD166-PE (clone REA442, Miltenyi Biotech GmbH); CD326-PE (clone HEA-125, Miltenyi Biotech GmbH); CD31-FITC (clone WM59, Dako, Agilent, Santa Clara, CA, USA); CD13-PE (clone WM15, BioLegend via Biozol GmbH); CD29PE (clone MAR4, BD Bioscience GmbH); CD44-FITC (clone G44-26, BD Bioscience GmbH); CA125 (clone X75, Abcam, Cambridge, UK); CD54-PE (clone HCD54, BioLegend via Biozol GmbH); CD106-PE (clone STA, BioLegend via Biozol GmbH); CD24-FITC (clone ML5, BD Bioscience GmbH); CD14-PE (clone TÜK4, Miltenyi Biotech GmbH); CD45-PE (clone HI30, BioLegend via Biozol GmbH); CD133-PE (clone293C3, Miltenyi Biotech GmbH); CD271-PE (clone ME20.4-1.H4, Miltenyi Biotech GmbH); mesothelin-PE (clone REA1057, Miltenyi Biotech GmbH). PE-, APC-, PB-, or FITC-labeled IgG antibodies of the corresponding IgG subclass (Dako) served as a control. Thereafter, the cells were washed again with PBS and subsequently analyzed by a flow cytometer using FACSCalibur (BD Biosciences GmbH) and FlowJo V10 software.

### 4.3. Senescence-Associated-β-galactosidase (SA-β-gal) Assay

The SA-β-gal assay was performed as described elsewhere [40]. Briefly, the different MSC cultures were fixed and stained against the SA-β-gal for 24 h/37 °C in the dark according to the assay kit protocol (Cell Signaling Technology Inc., Danvers, MA, USA). Following two washes with PBS the documentation of the differentially-stained cell cultures was performed by phase contrast microscopy and by quantification of colored cells.

### 4.4. Cell Cycle Analysis

Distribution of the various MSC populations throughout the different phases of the cell cycle was analyzed and quantified as described previously [41]. Briefly, 10^5^ cells were fixed in 70% (*v*/*v*) ice-cold ethanol at 4 °C for 24 h. Thereafter, the fixed cells were stained with propidium iodide staining solution (12.5µg/mL propidium iodide, 0.5% Triton-X-100 and 100 U/mL DNase-free RNase in PBS). Thereafter, the samples were analyzed in a FACSCalibur (BD Biosciences, Singapore) flow cytometer using the FlowJo V10 cell cycle software (FlowJo LLC, Ashland, OR, USA).

### 4.5. Transcript Analysis by RT-PCR

Total RNA was isolated from the tumor tissues and the organs using RNeasy Mini Kit (Qiagen, Hilden, Germany) according to the manufacturer’s instructions. One µg RNA was reverse transcribed into cDNA and amplified by polymerase chain reaction (PCR) as described previously [42]. Reactions were performed with following specific primers (customized by Eurofins MWG GmbH, Ebersberg, Germany):

SA-β-gal (739 bp) forward: 5´-AGG GAG TCC TTG AGC GAA AC-3´; reverse: 5´-AGG GAG GAT CTG TGA GGT TAG T-3´
Ki-67 (389 bp) forward: 5`-TAT CAA AAG GAG CGG GGT CG-3´; reverse: 5`-TTG AGC TTT TCT CAT CAG GGT CA-3′
survivin (357 bp) forward: 5´-GAC CAC CGC ATC TCT ACA TTC-3´; reverse: 5´-GTT CTT GGC TCT TTC TCT GTC C-3´
PCNA (722 bp) forward: 5`-CAT CAA CGA GGC CTG CTG GGA T-3´; reverse: 5`-CCT AAG ATC CTT CTT CAT CCT CG-3´
c-myc (575 bp) forward: 5`-TGG TGC TCC ATG AGG AGA CA-3´; reverse: 5`-GTG TTT CAA CTG TTC TCG TC-3´
hTERT (95 bp) forward: 5`-TGA CAC CTC ACC TCA CCC AC-3’; reverse: 5`CAC TGT CTT CCG CAA GTT CAC-3’
Fibronectin (439 bp) forward: 5`-AGC CGC CAC GTG CCA GGA TTA C-3’; reverse: 5’-CTT ATG GGG GTG GCC GTT GTG G-3
α-sm-actin (443 bp) forward: 5´-CCT CCC TTG AGA AGA GTT ACG-3´; reverse: 5´-GAG CAG GAA AGT GTT TTA GAA-3´
BCL-2 (246 bp) forward: 5`-GAG TGA CAG TGG ATT GCA T-3´; reverse: 5`-CAG AAT ATC AGC CAC CTC TT-3´
GAPDH (452 bp) forward: 5`-ACC ACA GTC CAT GCC ATC AC-3’; reverse: 5’-TCC ACC ACC CTG TTG CTG TA-3’
RUNX2 (127 bp) forward: 5’-TGG CAT CAA ACA GCC TC-3’; reverse: 5’-CTC ACG TCG CTC ATT TT-3’
COL1A1 (653 bp) forward: 5’-GCG ACC AAG GTG ACA GAG GCA TAA-3’; reverse: 5’-GTT CGG GCT GAT GTA CCA GTT CTT-3’
BGLAP (297 bp) forward: 5’-ATG AGA GCC CTC AGA CTC CTC-3’; reverse: 5’-CGG GCC GTA GAA GCG CCG ATA-3’
FABP4 (290 bp) forward: 5’-TAT GAA AGA AGT AGG AGT GGG C-3’; reverse: 5’-CCA CCA CCA GTT TAT CAT CCT C-3’
CEBPα (131 bp) forward: 5’-TGG ACA AGA ACA GCA ACG AGT A-3’; reverse: 5’-ATT GTC ACT GGT CAG CTC CAG-3’
PPARγ (700 bp) forward: 5’-AAG AGC TGA CCC AAT GGT TGC TGA-3’; reverse: 5’-GGA TCG AAA CTG GCA CCC TTG AAA-3’
β-actin (100 bp) forward: 5’-CAG GCT GTG CTA TCC CTG TA-3’; reverse: 5’-CAT ACC CCT CGT AGA TGG GC-3’
PCR products were separated on a 2% agarose gel as described [41] and visualized by GelRed staining (Biotium Inc., Fremont, CA, United States).

### 4.6. Immunoblot Analysis

Protein identification in cell lysates was performed as previously described [43]. Briefly, total cellular protein of different MSC populations was separated on a 10% polyacrylamide gel, transferred to a nitrocellulose membrane and incubated using a monoclonal rabbit anti p16^INK4A^ antibody (clone D3W8G; dilution 1:1000; Cell Signaling Technology, Leiden, Netherlands); a polyclonal rabbit anti-p53 antibody (clone 9282; dilution 1:1000; Cell Signaling Technology); a monoclonal rabbit anti-telomerase reverse transcriptase (hTERT) antibody (clone Y182; dilution 1:1000; Abcam plc, Cambridge, UK), and a monoclonal mouse anti-GAPDH (clone 6C5; dilution 1:200; Santa Cruz Biotechnology Inc., California, USA). Antibody detection was performed with peroxidase-linked secondary antibody and subsequent chemiluminescence.

### 4.7. Cytotoxicity Assay Against K562 Target Cells

Proliferating and confluent MSC544 populations were seeded in 24-well plates (Nunc, Wiesbaden, Germany) at a density of 1 × 10^5^ cells/cm^2^ and allowed to adhere, respectively. Peripheral blood mononuclear cells (PBMCs) were prepared from peripheral blood by Ficoll density gradient centrifugation as described elsewhere [44]. Unstimulated PBMCs were added and the MSC populations in direct contact at a ratio 10:1 in RPMI 1640 media (Biochrom, Berlin, Germany) supplemented with 10% FCS, 1mM L-glutamine, 50 U/mL penicillin, 0.5mM sodium pyruvate and 50 μg/mL streptomycin (R10) [45]. Following the co-culture, PBMCs as effector cells were harvested and incubated with ^51^Cr–labeled K652 target cells at different effector to target (E:T) ratios in R10 for four hours. Samples were centrifuged (300g /5 min) and released radioactivity (counts per minute) was determined from cell-free supernatants [46].

### 4.8. Mass Spectrometric (MS) Analysis

Proteins released into the supernatant were analyzed from steady state proliferating MSC544 and from confluent MSC544 after 189d of permanent culture. Briefly, about 3 × 10^6^ cells of both MSC544 populations in culture were washed 3x with PBS to remove supernatant proteins and incubated using MSC culture medium without serum in the incubator for 36h. The supernatants containing newly released proteins were harvested, centrifuged (3,185 g /10 min) and aliquots were shock-frozen in liquid N_2_ until preparation for MS analysis as described [47]. Thereafter, the prepared peptide samples were separated with a nano-flow ultra-high pressure liquid chromatography (LC) system and subsequent LC-MS analysis was performed according to a previous protocol [39]. 

### 4.9. In Vivo Studies

Animal research using athymic NMRI-Foxn1^nu^ mice was carried out by following the internationally recognized guidelines on animal welfare and was approved by the institutional licensing committee ref. # 33-42502-06/1178 on Sep. 12th, 2006. For the in vivo experiments, 5- to 6-week-old female mice were used. About 10^6^ MSC544 were subcutaneously injected into the flank of 5 athymic NMRI-Foxn1^nu^ mice, respectively, and the mice were monitored for potential tumor growth and/or unhealthy development.

## 5. Conclusions

The identification of MSC-like features suggested mesenchymal stroma-/stem-like properties in MSC544 with fibroblast-like morphology during confluency. The reproducible MSC544 characteristics enable the identification of potential developmental switches upon changes of the cellular environment. Moreover, the MSC544 cell model would allow further functional studies, which would also be relevant for a general discrimination of MSC mixtures, pericytes and fibroblasts, and an important categorization of heterogeneous mesenchymal stroma-/stem-like cells into distinct stromal cell types and subpopulations displaying stem cell-like properties.

Whereas no in vivo tumor development could be detected with MSC544, this spontaneously permanent growing cell line could also serve as an off-the-shelf translational model in regenerative medicine, although standards and regulations for these approaches still need to be established. Indeed, the reproducible capability of connective cell layer formation indicates that human MSC544 with potentially infinite growth properties are regulated by contact inhibition and display properties of tissue-like production and regeneration. Moreover, MSC544 resulting products could be used in perpetual and reproducible therapeutic approaches. 

## Figures and Tables

**Table 1:** Flow cytometry analysis of various expression markers was performed in 12 different human MSC populations, six derived from umbilical cord (UC) in passage (P) 0 to P3 (blue background), three from bone marrow (BM) in P2 to P4 (red background), three from placenta (PL) (green background) in P1 to P2, and compared to MSC544 in P22 (yellow background). Proteins at low to undetectable levels are displayed by a khaki-colored background.

Antibody, Labeling	UC-MSC 180816 P0	UC-MSC 270815 P0	UC-MSC 030816 P1	UC-MSC 151116 P1	UC-MSC 210611 P2	UC-MSC 040211 P3	MSC544 P22	BM-MSC84 P2	BM-MSC 161115 (#1) P3	BM-MSC 161115 (#2) P4	PL-MSC 250116 P1	PL-MSC 270116 P1	PL-MSC 210116 P2
**CD13-PE**	**99.9**	**100.0**	**88.8**	**99.2**	**100.0**	**100.0**	**98.0**	**99.8**	**99.7**	**99.9**	**100.0**	**100.0**	**100.0**
**CD29-PE**	**99.6**	**99.6**	**99.7**	**92.1**	**99.4**	**99.8**	**99.8**	**99.2**	**99.6**	**98.5**	**99.3**	**98.7**	**99.4**
**CD44-FITC**	**90.0**	**95.0**	**99.0**	**96.9**	**99.2**	**99.6**	**99.2**	**97.0**	**98.6**	**98.8**	**98.1**	**94.1**	**92.4**
**CD73-PE**	**93.2**	**99.7**	**98.5**	**98.5**	**99.1**	**99.8**	**99.1**	**99.5**	**98.7**	**97.2**	**94.2**	**98.3**	**74.9**
**CD90-PE**	**94.2**	**86.7**	**99.0**	**98.4**	**99.7**	**99.8**	**99.9**	**97.2**	**99.4**	**97.1**	**87.6**	**97.6**	**94.4**
**CD105-PE**	**94.2**	**99.4**	**98.4**	**99.3**	**98.2**	**99.4**	**98.7**	**99.1**	**97.9**	**99.2**	**95.9**	**91.1**	**93.9**
**CD166-PE**	**94.6**	**94.7**	**97.8**	**99.2**	**99.9**	**93.5**	**99.5**	**99.7**	**99.5**	**99.1**	**98.8**	**97.9**	**97.0**
**CA125**	**95.3**	**93.3**	**96.5**	**-**	**96.9**	**79.3**	**93.0**	**95.7**	**83.4**	**37.6**	**58.1**	**67.9**	**62.6**
**CD146-PE**	**87.3**	**86.6**	**94.0**	**80.6**	**91.8**	**90.6**	**3.4**	**93.4**	**83.6**	**34.2**	**81.5**	**97.0**	**80.4**
													
**CD54-PE**	**69.2**	**87.9**	**84.0**	**56.5**	**82.2**	**79.7**	**69.8**	**99.6**	**96.3**	**85.7**	**94.9**	**99.5**	**95.1**
**CD106-PE**	**18.3**	**27.5**	**4.8**	**27.2**	**23.5**	**7.9**	**1.8**	**44.5**	**94.4**	**55.7**	**43.6**	**7.2**	**50.5**
**Mesothelin-PE**	**21.6**	**5.2**	**7.6**	**0.6**	**14.1**	**7.0**	**29.2**	**63.5**	**48.4**	**10.0**	**17.0**	**41.6**	**17.0**
													
**CD24-FITC**	**3.5**	**2.1**	**0.2**	**8.5**	**0.7**	**0.6**	**3.5**	**2.0**	**8.8**	**2.9**	**3.4**	**4.8**	**0.3**
**CD31-FITC**	**0.1**	**0.1**	**0.2**	**0.7**	**0.0**	**0.1**	**1.6**	**0.1**	**0.1**	**0.1**	**0.0**	**0.1**	**0.0**
**CD14-PE**	**0.2**	**0.1**	**0.0**	**0.6**	**0.0**	**0.0**	**0.4**	**-**	**0.2**	**-**	**0.7**	**0.1**	**0.4**
**CD45-PE**	**0.9**	**2.6**	**1.5**	**0.4**	**0.2**	**0.4**	**1.5**	**-**	**0.8**	**-**	**0.3**	**1.3**	**0.1**
**CD133-PE**	**4.3**	**1.4**	**3.1**	**0.6**	**2.4**	**2.2**	**1.6**	**2.9**	**1.7**	**-**	**2.4**	**3.2**	**1.9**
**CD326-PE**	**2.7**	**1.5**	**0.8**	**0.6**	**0.6**	**1.9**	**0.2**	**-**	**0.3**	**-**	**0.6**	**0.9**	**0.5**
**CD295-PE**	**3.1**	**0.0**	**1.3**	**4.6**	**0.8**	**2.8**	**2.2**	**-**	**2.1**	**-**	**0.1**	**0.3**	**0.2**
**CD271-PE**	**1.3**	**1.5**	**0.9**	**0.4**	**1.0**	**1.8**	**0.7**	**2.5**	**6.4**	**9.0**	**0.7**	**0.5**	**0.6**

“#” represents different individual MSC of the same nomenclature; data indicating “-“ were not detected.

**Table 2:** Flow cytometry analysis of various detectable expression markers was performed in steady state MSC544 as compared to MSC544 cultured for 70d in confluency and 70d confluent MSC544 after re-culture for 1d in a sub-confluent environment (blue background). Proteins at low to undetectable levels are displayed by a khaki-colored background.

Antibody, Labeling	MSC544 (Steady State)	MSC544 (Confluency for 70d)	MSC544 (1d Re-Culture after Confluency for 70d)
CD13-PE	100.0	100.0	100.0
CD29-PE	99.9	99.9	99.7
CD44-FITC	99.5	99.5	98.3
CD166-PE	100.0	97.5	89.7
			
CD54-PE	25.4	91.3	77.3
			
CD106-PE	0.0	0.8	0.2
CD146-PE	0.4	0.2	0.6
CD24-FITC	1.1	0.1	0.0
CD31-FITC	0.4	0.0	0.0
CD14-PE	0.1	0.1	0.1
CD45-PE	0.2	0.0	0.0
CD133-PE	0.3	0.1	0.3
CD326-PE	0.1	0.0	0.0
CD295-PE	0.0	0.0	0.3
CD271-PE	0.3	0.0	0.1

## Supplementary Materials

The following are available online at https://www.mdpi.com/1422-0067/21/13/4752/s1, Figure S1: Differentiation capacity of MSC544 is demonstrated after a 21d culture in appropriate osteogenic, chondrogenic, or adipogenic differentiation medium as described [13]. Alizarin red S staining was performed after osteogenic maturation (**A**, upper 2 panels) together with a population overview (**A**, lower 2 panels). Further analysis of osteogenic-associated gene expression by PCR (**B**, upper panel) and quantification of relative expression levels after normalization to β-actin (**B**, lower panel) demonstrated little if any differences in RUNX2 expression. In contrast, alpha-1 type I collagen (COL1A1) and gamma-carboxyglutamic acid-containing protein (BGLAP/osteocalcin) were enhanced expressed in MSC544 after induction of osteogenic differentiation. Chondrogenic potential of MSC544 (C, upper 2 panels) together with a population overview (**C**, lower 2 panels) is demonstrated by Alcian blue staining. Adipogenic-associated gene expression by PCR (**D**, upper panel) and quantification of relative expression levels after normalization to β-actin (**D**, lower panel) revealed induction of the fatty acid-binding protein 4 (FABP4) and the transcription factor CCAAT/enhancer-binding protein alpha (CEBPα) but unchanged levels of peroxisome proliferator-activated receptor gamma (PPARγ). Bars represent 250µm; Figure S2: Differently-shaped GFP-labeled MSC544 in steady state culture (**A**) after long-term maintenance in confluency by changing the culture medium once a week developed a progressively dense population with a spindle fibroblast-like morphology between day 25 (**B**) and day 416 (**C**). Spontaneous contraction disrupted the cell layer of extracellular matrix-connected cells whereby distinct cells were maintained in the culture after 30sec of disruption (**C**, **D**, white arrows); various disruption bodies of damaged cells remained after contraction (**E**, yellow arrows) whereby the newly available space in the culture dish was subsequently filled by regained proliferative capacity of small cells from the previously growth-arrested MSC544 (**F**, **G**, white arrows); contraction of the cells layer can stop at some point due to multicellular strings connected with the rim of the culture dish (F, black arrows) or the contraction can eventually continue to form a globular body; Figure S3: Identification of MSC544 by a cell line authentication pattern was performed via short tandem repeat (STR) fragment analysis using the GenomeLab human STR primer set (Beckman Coulter Inc., Fullerton, CA, USA) as described previously [48]. The fragment analysis demonstrated an identical STR pattern of proliferating MSC544 P16 when compared to growth-arrested MSC544 P25 after 152d of continuous confluency.

## Abbreviations

FACS	Fluorescence-activated cell sorting
FCS	Fetal calf serum
GFP	Green fluorescent protein
hTERT	human telomerase reverse transcriptase
MSC	Mesenchymal stroma/stem-like cells
P	Passage
SA-β-gal	Senescence-associated beta-galactosidase
