# Supplementary material for: Reversible Growth-Arrest of a Spontaneously-Derived Human MSC-Like Cell Line
Source: Int J Mol Sci. 2020 Jul 3;21(13):4752. doi: 10.3390/ijms21134752 (PMC7369918; doi:10.3390/ijms21134752)
Supplement: Supplementary file 1 [file ijms-21-04752-s001.pdf]

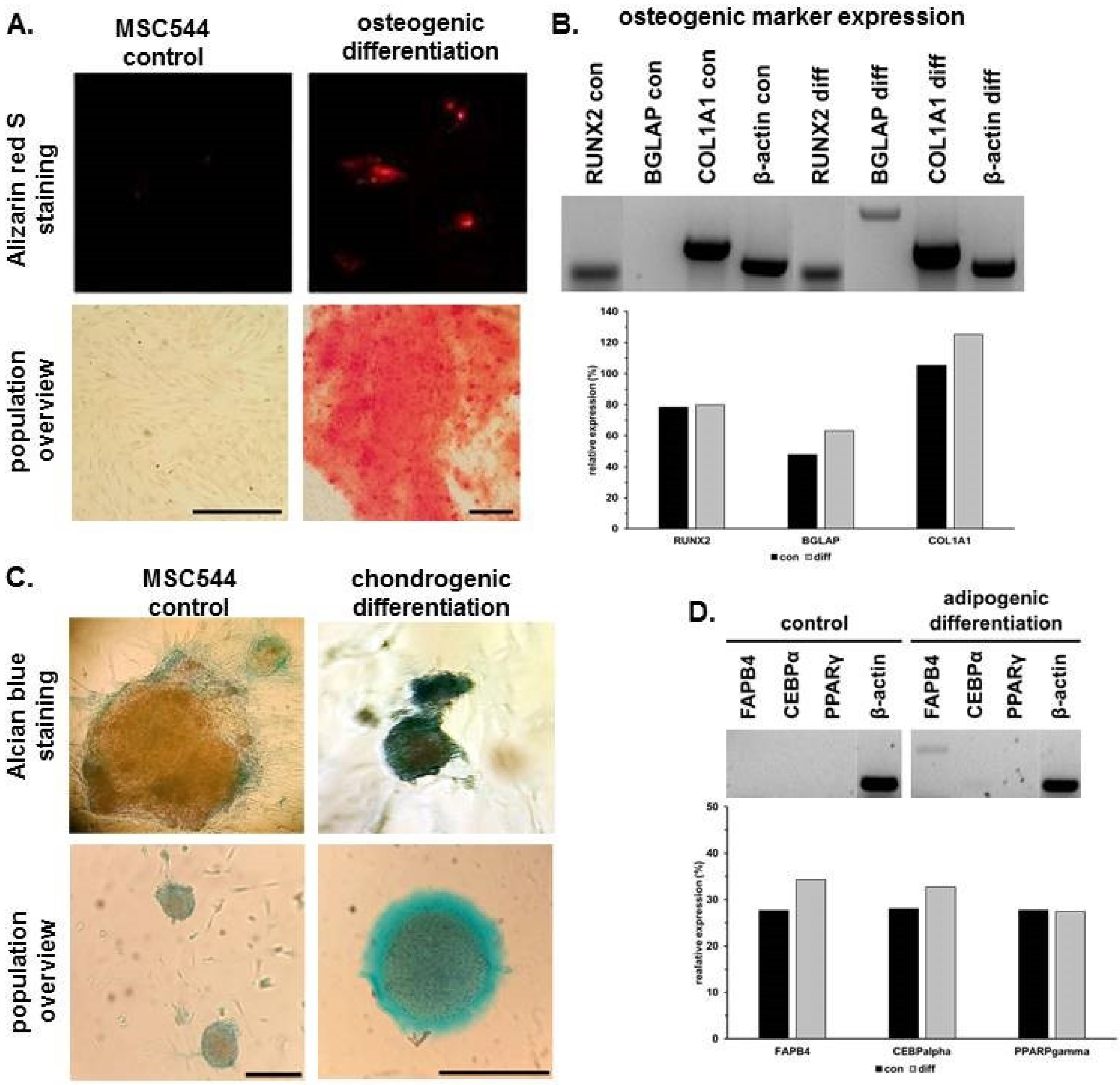

Figure S1. Differentiation capacity of MSC544 is demonstrated after a 21d culture in appropriate osteogenic, chondrogenic, or adipogenic differentiation medium as described [13]. Alizarin red S staining was performed after osteogenic maturation (**A**, upper 2 panels) together with a population overview (**A**, lower 2 panels). Further analysis of osteogenic-associated gene expression by PCR (**B**, upper panel) and quantification of relative expression levels after normalization to β-actin (**B**, lower panel) demonstrated little if any differences in RUNX2 expression. In contrast, alpha-1 type I collagen (COL1A1) and gamma-carboxyglutamic acid-containing protein (BGLAP/osteocalcin) were enhanced expressed in MSC544 after induction of osteogenic differentiation. Chondrogenic potential of MSC544 (C, upper 2 panels) together with a population overview (**C**, lower 2 panels) is demonstrated by Alcian blue staining. Adipogenic-associated gene expression by PCR (**D**, upper panel) and quantification of relative expression levels after normalization to β-actin (**D**, lower panel) revealed induction of the fatty acid-binding protein 4 (FABP4) and the transcription factor CCAAT/enhancer-binding protein alpha (CEBPα) but unchanged levels of peroxisome proliferator-activated receptor gamma (PPARγ). Bars represent 250μm.

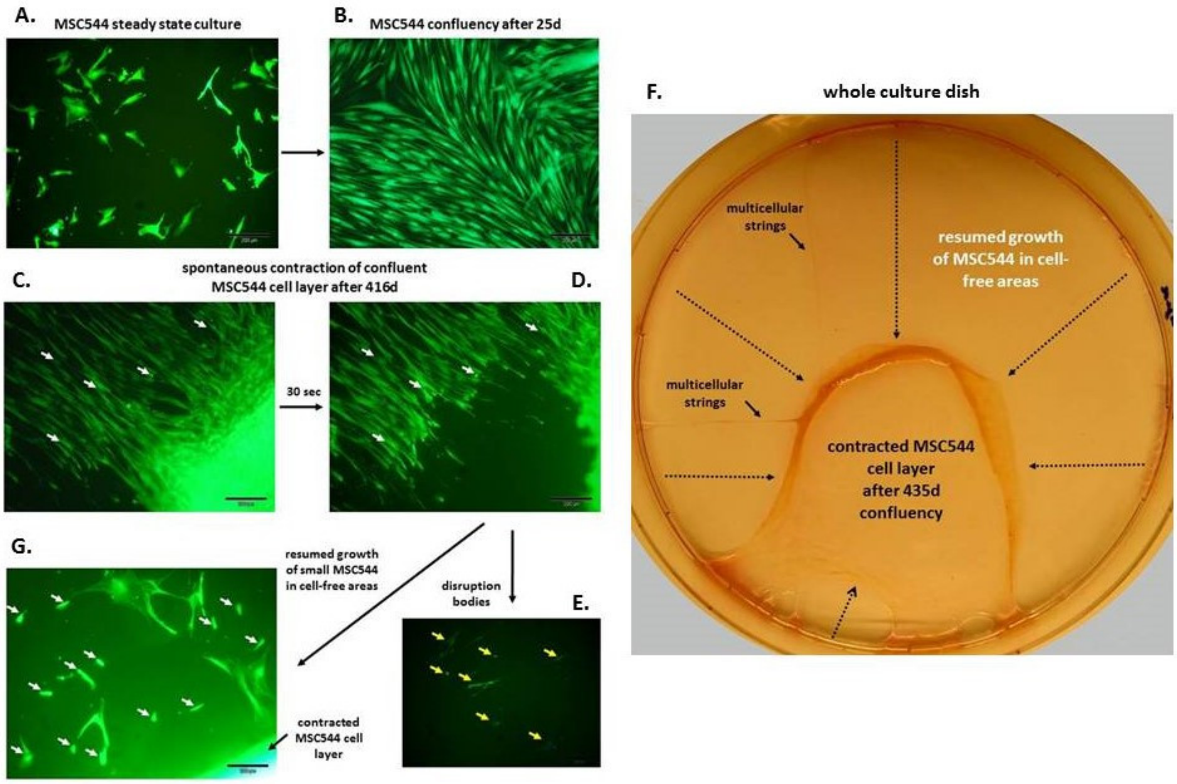

Figure S2. Differently-shaped GFP-labeled MSC544 in steady state culture (**A**) after long-term maintenance in confluency by changing the culture medium once a week developed a progressively dense population with a spindle fibroblast-like morphology between day 25 (**B**) and day 416 (**C**). Spontaneous contraction disrupted the cell layer of extracellular matrix-connected cells whereby distinct cells were maintained in the culture after 30sec of disruption (**C**, **D**, white arrows); various disruption bodies of damaged cells remained after contraction (**E**, yellow arrows) whereby the newly available space in the culture dish was subsequently filled by regained proliferative capacity of small cells from the previously growth-arrested MSC544 (**F**, **G**, white arrows); contraction of the cells layer can stop at some point due to multicellular strings connected with the rim of the culture dish (F, black arrows) or the contraction can eventually continue to form a globular body;.

STR fragment	MSC544 P16 allel 1	MSC544 P16 allel 2	MSC544 P25 after 152d of continuous confluency allel 1	MSC544 P25 after 152d of continuous confluency allel 2
Amelogenin	X-chrom.	X-chrom.	X-chrom.	X-chrom.
CSF1PO	9	12	9	12
D10S1248	14	16	14	16
D12S391	19	23	19	23
D13S317	11	11	11	11
D16S539	12	12	12	12
D18S51	13	15	13	15
D19S433	14	16	14	16
D1S1656	12	16	12	16
D21S11	28	30	28	30
D22S1045	15	16	15	16
D2S1338	16	17	16	17
D2S441	13	14	13	14
D3S1358	14	15	14	15
D7S820	8	9	8	9
D8S1179	9	13	9	13
FGA	19	25	19	25
SE33	15	29.2	15	29.2
TH01	6	6	6	6
VWA	15	16	15	16

Figure S3. Identification of MSC544 by a cell line authentication pattern was performed via short tandem repeat (STR) fragment analysis using the GenomeLab human STR primer set (Beckman Coulter Inc., Fullerton, CA, USA) as described previously [48]. The fragment analysis demonstrated an identical STR pattern of proliferating MSC544 P16 when compared to growth-arrested MSC544 P25 after 152d of continuous confluency.
